# Mid-Term Impact of Conduction System Pacing on Overall Cardiac Performance: A Non-Randomized, Prospective, Single-Center Echocardiographic Study

**DOI:** 10.3390/diseases12120321

**Published:** 2024-12-10

**Authors:** Catalin Pestrea, Ecaterina Cicala, Roxana Enache, Marcela Rusu, Radu Gavrilescu, Adrian Vaduva, Madalina Ivascu, Florin Ortan, Dana Pop

**Affiliations:** 1 Department of Interventional Cardiology, Brasov County Clinical Emergency Hospital, 500326 Brasov, Romania; cicalaecaterina@gmail.com (E.C.); spac.roxana@yahoo.com (R.E.); mmurafa@yahoo.ro (M.R.); radu.rgavrilescu@gmail.com (R.G.); tadi_vaduva@yahoo.co.uk (A.V.); madalinaclem@yahoo.com (M.I.); ortan.florin@gmail.com (F.O.); 2 5th Department of Internal Medicine, Faculty of Medicine, “Iuliu Hațieganu” University of Medicine and Pharmacy, 400012 Cluj-Napoca, Romania; pop67dana@gmail.com; 3 Department of Cardiology, Clinical Rehabilitation Hospital, 400347 Cluj-Napoca, Romania

**Keywords:** pacing-induced cardiomyopathy, heart failure, His bundle pacing, left bundle branch area pacing, mid-term follow-up

## Abstract

**Introduction.** Recently published data suggested significantly lower pacing-induced cardiomyopathy (PICM) incidence with conduction system pacing (CSP). Because most data evaluated only the impact on the left ventricle, this study aimed to assess changes in echocardiographic parameters of morphology and function for all heart chambers in patients with baseline preserved and mid-range LVEF over a medium-term follow-up period after CSP. **Methods.** A total of 128 consecutive patients with LVEF > 40% and successful CSP for bradyarrhythmic indication were prospectively enrolled. A complete 2D echocardiographic examination was performed at baseline and the last follow-up. **Results.** In total, 38 patients received His bundle pacing (HBP) and 90 received left bundle branch area pacing (LBBAP). The mean follow-up period was 699.2 ± 177.2 days, with 23 patients lost during this period. The ventricular pacing burden for the entire group was 97.2 ± 4.2%. Only three patients (2.9%) met the criteria for PICM. CSP led to a significant increase in LVEF (from 54.2 ± 7.9 to 56.7 ± 7.8%, *p* = 0.01) and a significant decrease in LV diastolic (from 107.2 ± 41.8 to 91.3 ± 41.8 mL, *p* < 0.001) and systolic (from 49.7 ± 21.4 to 39.5 ± 18.2 mL, *p* < 0.001) volumes. There were no significant changes in E/e′, mitral regurgitation, atrial volumes, and right ventricle (RV) diameter. There was a significant improvement in RV function. Tricuspid regurgitation was the only parameter that worsened. There were no differences in evolution for each echocardiographic parameter between the HBP and the LBBAP groups. **Conclusions.** HBP and LBBAP are equally protective for harmful changes in both atria and ventricles. The prevalence of PICM, defined as a decrease in LVEF, is very low with CSP.

## 1. Introduction

The increased interest in conducting system pacing (CSP) over the past two decades and the amount of data on this topic gathered so far, albeit mostly from non-randomized studies, supported the introduction of these procedures in the current pacing guidelines [1]. The two physiological pacing procedures, targeting the bundle of His (HB) or the left bundle branch area, emerged as possible solutions to the extensive evidence showing deleterious effects on left ventricular (LV) function with long-term conventional right ventricular pacing (RVP). This latter condition, termed pacing-induced cardiomyopathy (PICM), is believed to be secondary to the significant electrical and mechanical dyssynchrony associated with right ventricular (RV) myocardial pacing [2]. Previous studies have identified wide-paced QRS complexes, reduced baseline left ventricular ejection fraction (LVEF), and high ventricular pacing burden as major predictors of PICM in chronic RVP [3].

The advantage of CSP over RVP is fast ventricular depolarization through the intrinsic conduction system, which leads to synchronous activation of opposing LV walls and a significant reduction in dyssynchrony. Previous studies have proven the efficacy of CSP in preserving and even improving LVEF [4].

PICM is generally defined as a decrease in LVEF of more than 10% after pacing, resulting in a final value of less than 50%. Using this definition, some studies of RVP have reported a prevalence for PICM of up to 20% over a three-year follow-up period [5]. Conversely, recently published data suggested that the incidence of PICM with CSP is significantly lower [6].

Unfortunately, most studies have focused on LV systolic function, and little is known about the impact of medium-term CSP on the function and morphology of the right ventricle and the atria.

This study aimed to evaluate changes in echocardiographic parameters of morphology and function for all heart chambers in patients with baseline preserved and mid-range LVEF over a medium-term follow-up period after CSP.

## 2. Materials and Methods

### 2.1. Study Design

This was a non-randomized, prospective, single-center study.

### 2.2. Patient Selection

All consecutive patients with a successful physiological pacing procedure for symptomatic bradyarrhythmias (atrioventricular block, slow conducting atrial fibrillation, or sick sinus syndrome) between the 1 January 2021 and the 31 June 2022 in the Cardiac Pacing Laboratory of the Brașov County Clinical Emergency Hospital in Romania were eligible for enrollment in the study. The inclusion criteria were (1) aged over 18 years old; (2) proof of capture of the conduction system (see below); (3) a complete 2D echocardiographic study with adequate acoustic window; (4) an LVEF > 40%; and (5) willingness to return for follow-up visits. The exclusion criteria were (1) severe symptomatic valvular disease; (2) recent acute coronary syndrome (within six months); (3) loss of capture of the conduction system during follow-up visits; and (4) life expectancy less than one year due to comorbidities.

In the end, 128 patients, 38 with His bundle pacing (HBP) and 90 with left bundle branch area pacing (LBBAP), were included in the initial analysis. The patients’ baseline demographic and clinical characteristics were recorded.

### 2.3. Pacing Procedure

All CSP procedures used a C315 His or a C304 His catheter (Medtronic, Minneapolis, MN, USA) and a Select Secure 3830 lead (Medtronic, Minneapolis, MN, USA). In most cases, an initial attempt was made to identify the HB electrogram.

For HBP, the delivery kit was placed at the anteroseptal tricuspid annulus, and the HB electrogram was identified using unipolar mapping. Pacing at decremental amplitudes was performed to ascertain HB capture and to determine the HB capture threshold. We used the following definitions for successful HB capture [7]:

- Selective HB capture—identical QRS and ST-T patterns to the baseline morphology and an isoelectric line between the pacing artifact and the beginning of the QRS complex.
- Non-selective HB capture—a “pseudo delta wave” aspect at the beginning of the paced QRS complex instead of an isoelectric line and a clear transition in morphology with decremental pacing to either selective HB pacing or pure myocardial capture.
- In patients with baseline bundle branch block, successful HB capture was considered when correction (total or partial) of the bundle branch block occurred, with at least a 30% reduction in QRS duration.

Usually, a final capture threshold of below 1.5 to 2 V was accepted. The implanter could abandon the HBP procedure and switch to LBBAP at any time.

LBBAP was performed as the first option or as a bailout solution after initial failed HBP. The delivery kit was placed approximately 1.5 cm from the HB position towards the RV apex, where the lead was screwed deep into the septum.

To confirm the capture of the left-sided conduction system, we used the commonly employed criteria of QRS, or pseudo-RBBB morphology in lead V1, plus one of the following [8]:

- QRS transition from non-selective to selective pacing with decremental or programmed stimulation.
- The presence of a left bundle branch/fascicular potential.
- An LV activation time (measured as R wave peak time in lead V6) shorter than 80 ms for a baseline narrow QRS complex and shorter than 90 ms for a wide baseline QRS complex.
- A V6-V1 inter-peak interval of at least 33 ms in case of longer LV activation times.

In successful CSP cases, no ventricular backup leads were implanted. All patients in sinus rhythm received an atrial lead. The leads were connected to a pacemaker since none of the patients had a history of ventricular tachycardia, and the minimum ejection fraction was 40%. The devices were programmed in DDD or DDDR mode with slightly shorter AV delays in sinus rhythm and VVI or VVIR mode at a higher pacing rate (usually at 70 bpm) than the intrinsic rhythm in atrial fibrillation to ensure constant ventricular pacing.

All procedural-related parameters (paced QRS duration, impedance, pacing and sensing thresholds, fluoroscopy, and total procedural time) were recorded.

### 2.4. Echocardiography

The following echocardiographic parameters were measured by the same operator, according to the current recommendations, one day before the pacing procedure and at the end of the follow-up period: left atrial volume (LAV) using the disk summation algorithm, left ventricular end-diastolic volume (LVEDV), left ventricular end-systolic volume (LVESV), LVEF using the biplane method of disks (modified Simpson’s rule), LV outflow tract velocity time integral (LVOT VTI), E/A, E/e′, mitral regurgitation (MR), right atrial volume (RAV) using a single-plane method of disks, RV diameter, tricuspid annular plane systolic excursion (TAPSE), peak systolic velocity of the tricuspid annulus by pulsed-wave Tissue Doppler Imaging (RV S′ wave) and tricuspid regurgitation (TR) [9,10].

### 2.5. Follow-Up

The patients were followed prospectively in the outpatient clinic. At follow-up, pacemaker interrogation was performed with 12-lead monitoring to assess conduction system capture. The initial echocardiographic measurements were repeated in patients with confirmation of adequate capture (change in QRS morphology with decremental amplitude pacing or with extra-stimuli testing). PICM was defined as a decrease in LVEF by at least 10%, resulting in a final value below 50% in the absence of other cardiac factors that could have contributed to the decline.

### 2.6. Statistical Analysis

Continuous variables were presented as mean ± one standard deviation or as median and interquartile range, and categorical variables were presented as frequencies and percentages. The Shapiro–Wilk test assessed the normality of distribution for all groups. Statistical comparison of means was performed using the *t*-test or the Mann–Whitney U test for independent groups and the *t*-test or Wilcoxon test for dependent groups according to the normality of distribution. The Chi-squared test evaluated the statistical difference between percentages. The association between continuous and dichotomous variables was assessed using binomial logistic regression. For all tests, a confidence interval of 95% was used, and a *p* < 0.05 was considered statistically significant.

Statistical analysis was performed using SPSS software v 26.0 (IBM, Armonk, NY, USA).

### 2.7. Ethical Considerations

The study was approved by the institutional ethics committee and complied with all aspects of the Declaration of Helsinki.

All patients were informed and provided their written consent before the procedure.

## 3. Results

The baseline characteristics of the patients, overall and for each pacing strategy, are presented in Table 1. The median age was 71, and most patients were males (64.1%). Patients in the LBBAP group were younger than those in the HBP group. The main pacing indication was AV node dysfunction in the form of AV block (72.4%) and slow-conducting AF (17.9%). The baseline QRS was longer in the LBBAP group (134.6 ± 29.9 ms vs. 113.3 ± 27 ms, *p* < 0.001), and RBBB morphology was more frequently encountered. There were no differences regarding comorbidities and medical treatment, except for a higher prevalence of mineralocorticoid receptor antagonist treatment in patients with LBBAP. In patients with a baseline EF < 50%, the incidence of bundle branch block was 63.9%, significantly more than in those with baseline EF ≥ 50% (43.5%, *p* = 0.05).

The comparative procedural parameters are shown in Table 2. In the HBP group, the QRS decreased from 113.3 ± 27 to 107.1 ± 23.9 ms (*p* = 0.09), and in the LBBAP group, the QRS decreased from 134.6 ± 29.9 to 128.6 ± 14.7 ms (*p* = 0.07). HBP produced a significantly narrower QRS complex and had shorter fluoroscopy times than LBBAP. On the other hand, LBBAP was significantly superior for capture and sensing thresholds. There was no difference in total procedural duration. No significant procedural complications were noted.

**Figure 1:**
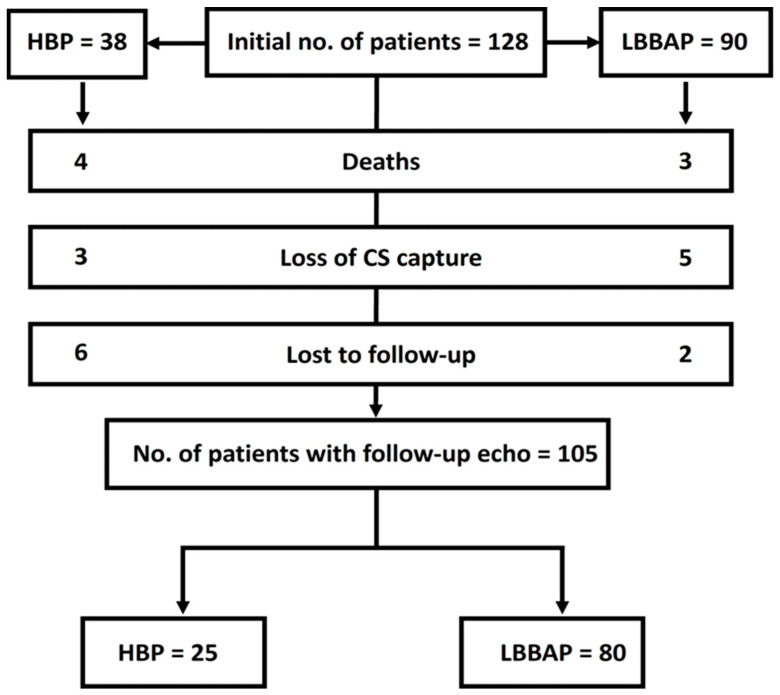
Study flow-chart. CS–conduction system; HBP–His bundle pacing; LBBAP–left bundle branch area pacing.

The mean follow-up period was 699.2 ± 177.2 days (minimum 397 days, maximum 1012 days). During this period, seven patients died due to non-cardiac-related causes (two oncological, three infectious, and two complications after general surgery), and another 16 patients lost conduction system capture or were lost to follow-up and could not provide final echocardiographic measurements. Therefore, 105 patients were included in the final analysis (Figure 1).

The follow-up pacing thresholds showed no significant change for either HBP (0.75 (0.5–1.1) V, *p* = 0.76) or LBBAP (0.5 (0.5–0.75) V, *p* = 0.96). Only one patient in each group had an increase of more than 1V in the pacing threshold. No lead macro-dislodgements were recorded.

Of the 105 patients, only three (2.9%) met the criteria for PICM: two in the LBBAP group and one in the HBP group. All of them had non-selective conduction system capture. The ventricular pacing burden for the entire group was 97.2 ± 4.2%.

**Figure 2:**
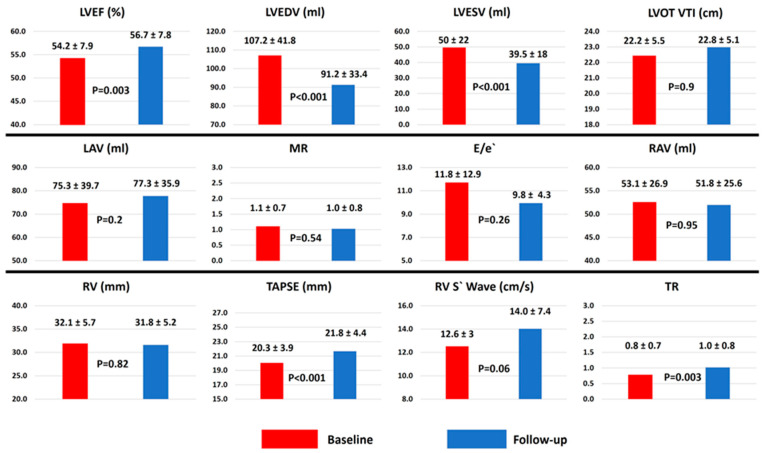
Comparison between baseline and follow-up echocardiography parameters for the entire study group. LVEF—left ventricular ejection fraction; LVEDV—left ventricular end-diastolic volume; LVESV—left ventricular end-systolic volume; LVOT VTI—left ventricular outflow tract velocity-time integral; LAV—left atrial volume; MR—mitral regurgitation; RAV—right atrial volume; RV—right ventricle; TAPSE—tricuspid annular plane systolic excursion; TR—tricuspid regurgitation.

The baseline and the follow-up echocardiographic values are presented in Figure 2. Overall, CSP led to a significant increase in LVEF and significant decreases in LVEDV and LVESV. At the end of the follow-up, there were no significant changes in E/e′, mitral regurgitation, atrial volumes, and RV diameter. There was a significant improvement in RV function, measured by TAPSE and RV S′ wave. The only parameter that worsened was tricuspid regurgitation, albeit from a mean of 0.8 to 1, but reaching statistical significance. Also, there were no significant differences in evolution for all echocardiographic parameters studied between patients with sinus rhythm and those with atrial fibrillation.

When considering the baseline LVEF, the overall improvement in LVEF was driven by the patients with initial values < 50%, who had an increase of 7.7 ± 7.3% compared to 0.3 ± 7.7% in the patients with initial values ≥ 50% (*p* < 0.001). The same conclusion was reached for the decrease in LVEDV (−31.1 ± 36.6 mL vs. −11.7 ± 23.8 mL, *p* = 0.01) and LVESV (−21.4 ± 21.6 mL vs. −6 ± 11.9 mL, *p* = 0.002). Also, 78.6% of patients with a baseline reduced LVEF showed improved RV function, compared to 50.6% of patients with baseline preserved LVEF.

There was no difference in evolution for each echocardiographic parameter between the HBP and the LBBAP group (Table 3). Although there was a trend for a reduction in RAV and RV diameter during follow-up with HBP, statistical significance was not reached.

## 4. Discussion

The main findings of the study can be summarized as follows: (1) The incidence of PICM in our study with CSP, according to the most commonly used criteria, was 2.9%; (2) CSP led to improvements in LV and RV function over a medium-term follow-up period, especially in patients with decreased baseline LVEF; (3) Over the same follow-up period, CSP preserves the values of atrial volumes, valvular regurgitations, and LV diastolic function; (4) There was no difference in echocardiography outcomes between HBP and LBBAP.

### 4.1. Procedural Outcomes

HBP started as the best physiological pacing option because it uses both bundle branches for fast and synchronous biventricular activation. The consequence is a much narrower QRS compared to other pacing modalities, a finding confirmed by our study [11]. Once LBBAP, which used the same delivery tools, was standardized and implemented in clinical practice, it became the preferred CSP method in many laboratories worldwide [12]. The main advantage of the procedure is a much wider target area compared to the thin structure of the His bundle, which significantly increases the success rate. Because the lead is placed deep into the ventricular septum, LBBAP is associated, as this study also shown, with excellent pacing and sensing thresholds.^13^ A limitation of all CSP procedures is the high fluoroscopy time [13]. In our research, HBP required fewer fluoroscopy minutes than LBBAP. This is explained by the initial attempt, at that time, to identify and capture the HB in many of the final LBBAP patients, thus adding a few more minutes to the total fluoroscopy time. Nevertheless, the total procedural time was similar between the two pacing strategies. The time allocated for testing conduction system capture was primarily responsible for the longer procedural times compared to fluoroscopy times.

### 4.2. Echocardiographic Outcomes

#### 4.2.1. Left Heart Chambers

PICM has been extensively studied in patients with RVP, and its prevalence differs from study to study and according to several definitions. For example, a meta-analysis of 18 studies reported a PICM prevalence of between 6 and 25%, with a pooled prevalence of 12% [14]. In our research, the prevalence of PICM was significantly lower at 2.9%, which is in line with what has been suggested by other studies with CSP [15].

A common feature of HBP and LBBAP is that most of the LV myocardium is activated through the left bundle branch. This leads to a short total LV activation time and prevents electrical and mechanical dyssynchrony of opposing ventricular walls. As presented in our study, this translates into preserving LVEF and LV volumes in patients with normal baseline LV function and anatomy. In a study by Vijayaraman et al. comparing HBP with RVP in patients with baseline normal LV function at follow-up after five years, they found that HBP preserved the LVEF (55 ± 8 vs. 57 ± 6%, *p* = 0.13) compared to RVP (57 ± 7% vs. 52 ± 11%, *p* = 0.002) and that the incidence of PICM, using the same definition as in our study, was 2% in the HBP vs. 22% in the RVP arm [15]. In another study, Li et al. showed that 235 patients with LBBAP had stable LVEF and slightly decreased LV end-diastolic diameter at the 1-year follow-up point (62.6 ± 4.6 vs. 61.7 ± 7.4% and 46.6 ± 5.2 vs. 49.4 ± 6.6 mm, respectively), compared to RVP [16]. Furthermore, CSP seems at least as effective as the current gold standard, biventricular pacing, for patients with developed PICM during RVP and the need to upgrade to one of the two therapies. In a meta-analysis including observational studies for CSP and both observational and randomized trials for biventricular pacing, LVEF increased after upgrade from RVP with 8.4% in the biventricular group compared to 11.1% in the LBBAP and 12.7% in the HBP group [17].

Our study showed that the subgroup of patients that benefited the most from physiological pacing were those with baseline mild to moderate reduced LVEF. This finding is in line with previous reports of similar patients. Bednarek et al. recently published a study on 101 patients with LBBAP and proved that patients with LVEF below 50% had an increase in LVEF from 41.4 ± 9.2 to 45.6 ± 9.9% (*p* < 0.001), while patients with normal baseline LVEF had similar values after follow-up at 23 months [6]. Also, Zhang et al. followed 64 CSP patients, including 16 LBBAP cases and 48 cases of HBP. After a mean of 23.12  ±  8.17 months follow-up time, LVEF (42.45  ±  1.84% vs. 49.97  ±  3.57%, *p*  <  0.001) and LV end-diastolic diameter (55.59  ±  6.17 mm vs. 51.66  ±  3.48 mm, *p*  <  0.001) improved significantly [18]. One possible explanation for the benefit in this category of patients is the higher prevalence of bundle branch block morphologies (63.9 vs. 43.5% in our study). It is widely known that intraventricular conduction anomalies cause functional impairment of the LV and that CSP is very effective in correcting these anomalies. Most evidence comes from patients with conventional cardiac resynchronization therapies for severely reduced LVEF and left bundle branch blocks (LBBBs). Two randomized studies evaluating the role of HBP in these patients showed non-inferiority compared to biventricular pacing regarding LV function improvement and demonstrated LBBB correction in 52% and 72% of the patients, respectively [19,20]. Right bundle branch block (RBBB) is also amenable to correction with CSP. In a study published by the International LBBAP Collaborative-Study Group, which included patients with RBBB and heart failure, LVEF improved in patients with baseline values between 36% and 50% from 42% ± 4% to 50% ± 8% (*p* < 0.01), and overall LVEDV decreased from 136 ± 61 to 126 ± 54 mL (*p* = 0.31) [21].

Although we agree that common sense dictates that there should be no pacing-induced cardiomyopathy with conduction system pacing, we believe there could be some explanations for the three patients in our study group. First, they all had non-selective capture of the conduction system, possibly with enough activation of the working myocardium to induce symptomatic dyssynchrony. Second, undiagnosed underlying subclinical cardiomyopathies or comorbidities could have accounted for the decline in LVEF over time, despite the protective effect of conduction system capture.

Few data on LV diastolic function in the long-term with CSP have been published. Theoretically, the physiological nature of depolarization and optimal LV mechanics with CSP should be followed by efficient repolarization and diastolic filling. In our study, the echocardiographic measurements of LV diastolic function (E/e′ and E/A) had a trend towards improvement but without reaching statistical significance, similarly to other reports in the literature [6].

An important finding was that left atrial volume and mitral regurgitation did not change over time. Early studies with conventional RVP found an increased incidence of atrial fibrillation in patients with high ventricular pacing burden [22]. One of the mechanisms involved was left atrial remodeling as a consequence of progressive LV impairment. Although few studies have looked explicitly at atrial changes, there are indirect data on the benefits of both HBP and LBBAP for atrial function and morphology proving significantly fewer episodes of new-onset atrial fibrillation with these procedures than RVP [23,24].

The direct comparison between the changes observed with HBP and LBBAP on the left heart echocardiographic parameters revealed no significant difference over a mid-term follow-up period. We believe the answer resides in the fact that the two procedures share the same physiological pattern of LV depolarization through the left bundle branch, with all its positive consequences.

#### 4.2.2. Right Heart Chambers

HBP activates the RV by directly capturing the right bundle branch and has the advantage of synchronizing both ventricles for optimal myocardial workload. On the other hand, the activation pathway of the RV with LBBAP is less understood. Two mechanisms of RV depolarization during LBBAP have been postulated: direct transseptal activation from the lead tip and retrograde conduction up the left bundle, followed by anterograde conduction down the right bundle branch [25]. The pseudo-RBBB morphology achieved with LBBAP suggests a delay in RV activation, a possible cause for future RV function decline due to iatrogenic interventricular dyssynchrony. Fortunately, despite these concerns, we found that overall CSP preserved the right atrium and ventricle dimensions and was associated with improved RV function evaluated with TAPSE and RV S′ wave measurement. Furthermore, there were no significant differences in the impact of the two physiological pacing strategies on the right heart anatomy and function over time. However, a discrete trend for the better with HBP was noted (non-significant lower right atrial and RV dimensions after the follow-up period), possibly suggesting the superiority of direct activation of the right bundle branch. Besides physiological electrical activation, interventricular mechanical dependence plays an important role in better RV function. This was supported by the observation that patients with lower baseline LVEF more often showed an improvement in RV systolic parameters with increased LVEF after the procedure. Our results are supported by a recently published study in which Tian et al. evaluated RV function with 3D echocardiography in 65 patients with LBBAP. After a 6-month follow-up period, LBBAP significantly improved RV volumes and RVEF in patients with initial RV dysfunction [26].

The only parameter that worsened in our study was TR, although it did not lead to significant regurgitation. This has been a consistent finding in previous studies, with the primary mechanism involved being the impingement of the septal leaflet by the LBBAP lead and the HBP lead placed on the ventricular side of the tricuspid annulus [27].

### 4.3. Strengths and Limitations

To our knowledge, this was the first study to evaluate the impact of CSP on changes in echocardiographic measurements for all the heart chambers over a medium-term follow-up period and to compare HBP and LBBAP for these outcomes. Another feature of this study was the patient exposure to very high percentages of ventricular pacing, thus increasing the strength of our evidence.

However, certain limitations should be mentioned. This was a non-randomized, prospective, single-center study with a moderate number of patients. The study was not powered to assess robust endpoints like mortality or heart failure hospitalizations. For these, larger randomized trials should be conducted. Nevertheless, since PICM was extensively studied for RVP, we believe that having no comparison group for our CSP patients does not significantly diminish the value of our findings. Secondly, the doctor performing echocardiography was not blinded to pre- and postprocedural examinations, and newer techniques like 3D echocardiography may have been more accurate in determining chamber dimensions. Also, innovative echocardiographic data, such as those derived from speckle tracking echocardiography, could have provided important information concerning myocardial fibrosis and the effects of CSP procedures on intraventricular dyssynchrony by measuring the time-to-peak strain [28,29]. Lastly, a more extended follow-up period is necessary to accurately assess the impact of CSP on the different heart chambers’ anatomy and function compared to RVP. Unfortunately, this is not easy to achieve due to the recent adoption of physiological pacing techniques as standard procedures in hospitals worldwide. Except for very early adopters, in most laboratories, between the time needed for the learning curve and the build-up of patients, the follow-up times are shorter compared to RVP.

## 5. Conclusions

In patients with preserved LVEF, HBP and LBBAP are equally protective for harmful changes in the atria and ventricles after exposure to high ventricular pacing burden. The prevalence of PICM, defined as a decrease in LVEF, is very low with CSP. Patients with a baseline mid-range EF have a particular benefit in the medium term, showing significant improvement in LV function.

## Figures and Tables

**Table 1:** Baseline patient characteristics.

BASELINE CHARACTERISTICS	All	HBP	LBBAP	*p* Value
Number of patients	128	38	90	
Age (years, median (IQR))	71 (66–77)	75.5 (67.7–78)	70 (66–76.2)	0.02
Male (N, %)	82 (64.1)	26 (68.4)	56 (62.2)	0.55
BMI (Kg/m^2^, median (IQR))	27.8 (25.5–32.5)	27.7 (24.6–32.6)	27.9 (25.6–32.5)	0.27
eGFR (mL/min, median (IQR))	61.2 (52–74.1)	57.5 (50–70.7)	62.8 (52.1–74.3)	0.47
LVEF (%, mean ± SD)	54.2 ± 7.9	54.9 ± 8.4	53.9 ± 7.7	0.37
LVEF < 50% (N, %)	36 (28.1)	10 (26.3)	26 (28.8)	0.83
PACING INDICATION				
AV block (N, %)	95 (74.2)	26 (68.4)	69 (76.6)	0.37
Slow conducting AF (N, %)	23 (17.9)	8 (21)	15 (16.6)	0.61
Sick sinus syndrome (N, %)	10 (7.8)	4 (10.5)	6 (6.7)	0.48
BASELINE QRS				
QRS duration (ms, mean ± SD)	127.8 ± 30.6	113.3 ± 27	134.6 ± 29.9	<0.001
Normal QRS (N, %)	65 (50.8)	25 (65.8)	40 (44.4)	0.03
LBBB (N, %)	29 (22.7)	7 (18.4)	22 (24.4)	0.49
RBBB (N, %)	31 (24.2)	5 (13.2)	26 (28.9)	0.07
NIVCD (N, %)	3 (2.3)	1 (2.6)	2 (2.2)	1
COMORBIDITIES				
Hypertension (N, %)	110 (85.9)	33 (86.8)	77 (85.6)	1
Diabetes mellitus (N, %)	38 (29.7)	10 (26.3)	28 (31.1)	0.67
Ischemic disease (N, %)	37 (28.9)	12 (31.6)	25 (27.8)	0.67
Renal failure (N, %)	34 (26.6)	7 (18.4)	27 (30)	0.19
Persistent AF (N, %)	29 (22.7)	8 (21.1)	21 (23.3)	1
TREATMENT				
RAAS antagonists (N, %)	110 (85.9)	33 (86.8)	77 (85.6)	1
Beta-blockers (N, %)	95 (74.2)	29 (76.3)	66 (73.3)	0.82
MRAs (N, %)	32 (25)	4 (10.5)	28 (31.1)	0.01
Anticoagulants (N, %)	53 (41.4)	16 (42.1)	37 (42)	1

HBP—His bundle pacing; LBBAP—left bundle branch area pacing; IQR—interquartile range; BMI—body mass index; SD—standard deviation; eGFR—estimated glomerular filtration rate; LVEF—left ventricular ejection fraction; AV—atrioventricular; AF—atrial fibrillation; LBBB—left bundle branch block; RBBB—right bundle branch block; NIVCD—non-specified intraventricular conduction delay; RAAS—renin–angiotensin–aldosterone system; MRAs—mineralocorticoid receptor antagonists.

**Table 2:** Comparative procedural characteristics of HBP and LBBAP.

	HBP N = 38	LBBAP N = 90	*p* Value
Paced QRS duration (ms)	107.1 ± 23.9	128.6 ± 14.7	<0.001
Capture threshold (V)	1 (0.6–1.3)	0.5 (0.5–1)	0.001
Capture threshold (ms)	1	0.4	
R wave detection (mV)	3 (2.2–5)	10 (6.5–12)	<0.001
Pacing impedance (Ohm)	434 (369–490)	586 (486–684)	<0.001
Fluoroscopy time (min)	4.9 (3.3–7.5)	7.5 (5.4–12)	<0.001
Procedural duration (min)	111.2 ± 27.6	112.8 ± 25.5	0.52

HBP–His bundle pacing; LBBAP–left bundle branch area pacing.

**Table 3:** Comparative analyses of the changes in different echocardiographic parameters for HBP and LBBAP.

DIFFERENCE BETWEEN FOLLOW-UP AND BASELINE VALUES			
ECHO PARAMETERS	HBP N = 25	LBBAP N = 80	*p* Value
LVEF	0.6 ± 9.9	2.8 ± 7.6	0.23
LVEDV	−23.5 ± 25.7	−14.6 ± 29.6	0.18
LVESV	−13.1 ± 21.3	−8.9 ± 14.4	0.19
LVOT VTI	−0.3 ± 7.5	−0.5 ± 3.94	0.93
LAV	7.1 ± 33.7	2.6 ± 23.9	0.55
MR	−0.24 ± 0.8	0 ± 0.8	0.15
E/e′	0.3 ± 3.6	−2.9 ± 14.9	0.48
E/A	0.2 ± 0.2	−0.1 ± 1.1	0.42
RAV	−6.6 ± 17.7	1.9 ± 21.5	0.06
RV diameter	−1.9 ± 4.5	−0.2 ± 6.3	0.07
TAPSE	1.5 ± 3.4	1.4 ± 4.1	0.85
RV S′ wave	1 ± 3.7	1.7 ± 8.6	0.42
TR	0.2 ± 0.8	0.3 ± 0.8	0.66

HBP—His bundle pacing; LBBAP—left bundle branch area pacing; LVEF—left ventricular ejection fraction; LVEDV—left ventricular end-diastolic volume; LVESV—left ventricular end-systolic volume; LVOT VTI—left ventricular outflow tract velocity-time integral; LAV—left atrial volume; MR—mitral regurgitation; RAV—right atrial volume; RV—right ventricle; TAPSE—tricuspid annular plane systolic excursion; TR—tricuspid regurgitation.

## Data Availability

The data included in this article are available upon reasonable request.

## References

[1] Chung M.K., Patton K.K., Lau C.P., Dal Forno A.R.J., Al-Khatib S.M., Arora V., Birgersdotter-Green U.M., Cha Y.M., Chung E.H., Cronin E.M. (2023). 2023 HRS/APHRS/LAHRS guideline on cardiac physiologic pacing for the avoidance and mitigation of heart failure. J. Arrhythm..

[2] Cho S.W., Gwag H.B., Hwang J.K., Chun K.J., Park K.M., On Y.K., Kim J.S., Park S.J. (2019). Clinical features, predictors, and long-term prognosis of pacing-induced cardiomyopathy. Eur. J. Heart Fail..

[3] Elder D.H., Lang C.C., Choy A.M. (2011). Pacing-induced heart disease: Understanding the pathophysiology and improving outcomes. Expert Rev. Cardiovasc. Ther..

[4] González-Matos C.E., Rodríguez-Queralto O., Záraket F., Jiménez J., Casteigt B., Vallès E. (2024). Conduction System Stimulation to Avoid Left Ventricle Dysfunction. Circ. Arrhythm Electrophysiol..

[5] Khurshid S., Epstein A.E., Verdino R.J., Lin D., Goldberg L.R., Marchlinski F.E., Frankel D.S. (2014). Incidence and predictors of right ventricular pacing-induced cardiomyopathy. Heart Rhythm.

[6] Bednarek A., Kiełbasa G., Moskal P., Ostrowska A., Bednarski A., Sondej T., Kusiak A., Rajzer M., Jastrzębski M. (2023). Left bundle branch area pacing prevents pacing induced cardiomyopathy in long-term observation. Pacing Clin. Electrophysiol..

[7] Vijayaraman P., Dandamudi G., Zanon F., Sharma P.S., Tung R., Huang W., Koneru J., Tada H., Ellenbogen K.A., Lustgarten D.L. (2018). Permanent His bundle pacing: Recommendations from a Multicenter His Bundle Pacing Collaborative Working Group for standardization of definitions, implant measurements, and follow-up. Heart Rhythm.

[8] Jastrzębski M., Kiełbasa G., Cano O., Curila K., Heckman L., De Pooter J., Chovanec M., Rademakers L., Huybrechts W., Grieco D. (2022). Left bundle branch area pacing outcomes: The multicentre European MELOS study. Eur. Heart J..

[9] Lang R.M., Badano L.P., Mor-Avi V., Afilalo J., Armstrong A., Ernande L., Flachskampf F.A., Foster E., Goldstein S.A., Kuznetsova T. (2015). Recommendations for cardiac chamber quantification by echocardiography in adults: An update from the American Society of Echocardiography and the European Association of Cardiovascular Imaging. Eur. Heart J. Cardiovasc. Imaging.

[10] Nagueh S.F., Smiseth O.A., Appleton C.P., Byrd B.F., Dokainish H., Edvardsen T., Flachskampf F.A., Gillebert T.C., Klein A.L., Lancellotti P. (2016). Recommendations for the Evaluation of Left Ventricular Diastolic Function by Echocardiography: An Update from the American Society of Echocardiography and the European Association of Cardiovascular Imaging. Eur. Heart J. Cardiovasc. Imaging.

[11] Tokavanich N., Prasitlumkum N., Mongkonsritragoon W., Cheungpasitporn W., Thongprayoon C., Vallabhajosyula S., Chokesuwattanaskul R. (2021). A network meta-analysis and systematic review of change in QRS duration after left bundle branch pacing, His bundle pacing, biventricular pacing, or right ventricular pacing in patients requiring permanent pacemaker. Sci. Rep..

[12] Keene D., Anselme F., Burri H., Pérez Ó.C., Čurila K., Derndorfer M., Foley P., Gellér L., Glikson M., Huybrechts W. (2023). Conduction system pacing, a European survey: Insights from clinical practice. Europace.

[13] Vijayaraman P., Patel N., Colburn S., Beer D., Naperkowski A., Subzposh F.A. (2022). His-Purkinje Conduction System Pacing in Atrioventricular Block: New Insights Into Site of Conduction Block. JACC Clin. Electrophysiol..

[14] Somma V., Ha F.J., Palmer S., Mohamed U., Agarwal S. (2023). Pacing-induced cardiomyopathy: A systematic review and meta-analysis of definition, prevalence, risk factors, and management. Heart Rhythm.

[15] Vijayaraman P., Naperkowski A., Subzposh F.A., Abdelrahman M., Sharma P.S., Oren J.W., Dandamudi G., Ellenbogen K.A. (2018). Permanent His-bundle pacing: Long-term lead performance and clinical outcomes. Heart Rhythm.

[16] Li X., Zhang J., Qiu C., Wang Z., Li H., Pang K., Yao Y., Liu Z., Xie R., Chen Y. (2021). Clinical Outcomes in Patients With Left Bundle Branch Area Pacing vs. Right Ventricular Pacing for Atrioventricular Block. Front. Cardiovasc. Med..

[17] Kaza N., Htun V., Miyazawa A., Simader F., Porter B., Howard J.P., Arnold A.D., Naraen A., Luria D., Glikson M. (2023). Upgrading right ventricular pacemakers to biventricular pacing or conduction system pacing: A systematic review and meta-analysis. Europace.

[18] Zhang D.D., Zhao F.L., Yang Y.H., Ma C.M., Ma P.P., Zhao Y.N., Xia Y.L., Gao L.J., Dong Y.X. (2023). Conduction system pacing improves the outcomes on patients with high percentage of ventricular pacing and heart failure with mildly reduced ejection fraction. Front. Cardiovasc. Med..

[19] Upadhyay G.A., Vijayaraman P., Nayak H.M., Verma N., Dandamudi G., Sharma P.S., Saleem M., Mandrola J., Genovese D., Oren J.W. (2019). On-treatment comparison between corrective His bundle pacing and biventricular pacing for cardiac resynchronization: A secondary analysis of the His-SYNC Pilot Trial. Heart Rhythm.

[20] Vinther M., Risum N., Svendsen J.H., Møgelvang R., Philbert B.T. (2021). A Randomized Trial of His Pacing Versus Biventricular Pacing in Symptomatic HF Patients With Left Bundle Branch Block (His-Alternative). JACC Clin. Electrophysiol..

[21] Vijayaraman P., Cano O., Ponnusamy S.S., Molina-Lerma M., Chan J.Y.S., Padala S.K., Sharma P.S., Whinnett Z.I., Herweg B., Upadhyay G.A. (2022). Left bundle branch area pacing in patients with heart failure and right bundle branch block: Results from International LBBAP Collaborative-Study Group. Heart Rhythm O2.

[22] Nielsen J.C., Kristensen L., Andersen H.R., Mortensen P.T., Pedersen O.L., Pedersen A.K. (2003). A randomized comparison of atrial and dual-chamber pacing in 177 consecutive patients with sick sinus syndrome: Echocardiographic and clinical outcome. J. Am. Coll. Cardiol..

[23] Takahashi M., Kujiraoka H., Arai T., Kimura T., Hojo R., Fukamizu S. (2023). New-onset atrial high-rate episodes between his bundle pacing and conventional right ventricular septum pacing in patients with atrioventricular conduction disturbance. J. Interv. Card. Electrophysiol..

[24] Zhu H., Li X., Wang Z., Liu Q., Chu B., Yao Y., Liu Z., Xie R., Fan X. (2023). New-onset atrial fibrillation following left bundle branch area pacing vs. right ventricular pacing: A two-centre prospective cohort study. Europace.

[25] Jastrzębski M., Burri H., Kiełbasa G., Curila K., Moskal P., Bednarek A., Rajzer M., Vijayaraman P. (2022). The V6-V1 interpeak interval: A novel criterion for the diagnosis of left bundle branch capture. Europace.

[26] Tian F., Weng H., Liu A., Liu W., Zhang B., Wang Y., Cheng Y., Cheng S., Fulati Z., Zhou N. (2023). Effect of left bundle branch pacing on right ventricular function: A three-dimensional echocardiography study. Heart Rhythm.

[27] Li X., Zhu H., Fan X., Wang Q., Wang Z., Li H., Tao J., Wang H., Liu Z., Yao Y. (2022). Tricuspid regurgitation outcomes in left bundle branch area pacing and comparison with right ventricular septal pacing. Heart Rhythm.

[28] Sonaglioni A., Nicolosi G.L., Rigamonti E., Lombardo M., La Sala L. (2022). Molecular Approaches and Echocardiographic Deformation Imaging in Detecting Myocardial Fibrosis. Int. J. Mol. Sci..

[29] Pujol-López M., Jiménez-Arjona R., Garcia-Ribas C., Borràs R., Guasch E., Regany-Closa M., Graterol F.R., Niebla M., Carro E., Roca-Luque I. (2024). Longitudinal comparison of dyssynchrony correction and ‘strain’ improvement by conduction system pacing: LEVEL-AT trial secondary findings. Eur. Heart J. Cardiovasc. Imaging.

